# Will Australians Eat Alternative Proteins?

**DOI:** 10.3390/foods14091526

**Published:** 2025-04-26

**Authors:** Diana Bogueva, Dora Marinova

**Affiliations:** Curtin University Sustainability Policy (CUSP) Institute, Curtin University, Perth, WA 6845, Australia; d.marinova@curtin.edu.au

**Keywords:** alternative proteins, consumer perception, food safety, nutritional value, health risks, plant-based foods, cultured meat, insect-based proteins, algae-derived foods, Generation Z, food choices, Australia, food consumption, sustainability

## Abstract

Australia, which has one of the world’s highest per capita meat consumption rates, is hesitant toward adopting alternative proteins. This study examines consumer perceptions of protein alternatives and their perceived safety, nutritional value, health risks, cost and willingness to incorporate them into their diets. Using a mixed-methods approach, a survey of 520 Australians aged 18 to 64 revealed that while alternative proteins are viewed as occasional dietary options, younger consumers—despite their tendency to favour traditional food choices—show interest in plant-based milk and algae-based products. However, across all generations, interest in regular consumption of plant-based meats, insects and cultured meat remains low. These findings highlight evolving attitudes, challenges to market growth, and the importance of fostering greater consumer trust to encourage wider adoption of alternative proteins. Addressing generational differences in adoption and resistance will be essential for expanding market penetration.

## 1. Introduction

Traditional meat production has significant environmental consequences, including greenhouse gas emissions, land use and water consumption [1]. Alternative proteins such as plant-based proteins (including traditional soy, lentils and chickpeas, and non-traditional plant-based meats), cultured meat, algae (including in popular foods like sushi, snacks, and new applications in meat alternatives), and insect-based proteins are presented as offering a more sustainable way to meet our protein needs [2].

The food industry has been quick in responding to these opportunities with a wide range of products reaching consumers across the globe with support from private investors and government schemes [3]. This industry interest has been particularly pronounced in Europe and USA where environmental concerns are interwoven with opportunities to provide non-livestock protein sources to the growing global population and younger consumers [4]. Specifically in Australia, the alternative proteins industry is experiencing an evolving and growing trend, but its success has been uneven across the retail and food services sectors [5]. This is despite an increasing number of consumers reducing their meat intake, with health and environmental concerns being the top motivators [6].

Consumer attitudes toward alternative proteins shape not only individual dietary choices but also reflect a growing awareness of health, sustainability and food safety. Food choices are also deeply intertwined with culture, tradition and identity. By understanding and positively influencing consumer attitudes, it may be possible to pave the way for the successful integration of alternative proteins into everyday diets and foster broader societal shifts toward a more sustainable food consumption. Previous studies have explored the consumer landscape in terms of describing quantitatively Australian attitudes toward alternative proteins and have identified a large section of the Australian population, namely 43%, following flexitarian, meat reducer, vegan and vegetarian diets, as well as the main motivating factors for them to do so [6]. However, a greater share of Australians is reluctant to reduce the consumption of livestock-based products and/or replace them with alternative proteins. It is important to understand why this is the case and what concerns drive their attitudes.

The study presented in this paper helps provide some answers that can inform industry and government decisions. Alternative proteins continue to be an emerging area of research and industry efforts, and if they are to deliver widespread benefits, we need to understand consumer attitudes and address any concerns they have to overcome potential barriers and challenges. Using the mixed-methods methodology described below, the study’s aim was to collect and analyse numerical and exploratory data to gain a comprehensive understanding of Australian consumer attitudes toward the safety and nutritional value of alternative proteins.

Australia has one of the highest per capita meat consumption rates in the world, and although recently there has been slight replacement of beef with chicken in people’s preferences, the country is yet to experience a significant reduction in livestock-based meat choices [7]. There is already a wide range of plant-based protein alternatives on the Australian market. In addition to the more traditional tofu and soy milk products, consumers can now find vegan sausages, burgers, patties, cheese and ready meals that imitate the taste and texture of livestock-sourced products [8]. They are offered by international (e.g., Beyond Meat, Oatly and Vitasoy) and domestic (e.g., Made with Plants, So Good and v2food) brands supported by a surge in investment and government initiatives [8]. Despite progress in the processing and manufacturing capabilities, the market success of the alternative proteins has stagnated [9,10], and any future increases will largely depend on the consumers’ attitudes, experiences and expectations.

Australian consumers are becoming gradually aware of the environmental footprint of their food choices, with increasing interest in environmental labelling [11]. However, consumers also demand to know the healthfulness of the food they eat [12]. Alternative proteins are not an exception, and a better environmental performance is unlikely to be offset by inferior health and nutritional profile. Many plant-based proteins are considered ultra-processed, which may discourage consumers from eating them, but evidence suggests that certain processing may, in fact, improve their nutritional value by adding or augmenting the bioavailability of key nutrients [13,14].

The remainder of the paper sheds some light as to Australian consumers’ attitudes toward alternative proteins. We first explain the methodology of the study, then describe the survey sample, and present the results. The survey instrument allowed the participants to provide free-text answers about what they thought about different types of alternative proteins, which we were able to categorise into different groups of concerns. These concerns include health-related unease (food safety, processing and other health risks), nutritional value (nutritional deficiency, high sodium content and occasional consumption), consumer attitudes (transparency, masculinity and generational attitudes), environmental and ethical considerations. The discussion of the findings from the survey indicates that Australian consumers are not yet ready to accept the alternative proteins, with negative perceptions dominating their attitudes. Although there have been previous studies analysing Australian consumers [15,16], they have not covered some important themes that emerged from this investigation. Such themes include generational differences, need for transparency in the industry, and threats to traditional masculine ideas about (red) meat consumption and masculinity. Together with the wider discussed issues of food safety, nutritional value, additives and environmental benefits, the new themes offer a more nuanced and realistic perspective about the acceptability of alternative proteins in Australia.

## 2. Materials and Methods

A mixed-methods approach that combines qualitative and quantitative research methods in a single study [17] was adopted in this investigation, whose aim was to explore Australian consumers’ attitudes toward alternative proteins outside of their environmental benefits. Such an approach allows to better understand the issue by capturing different perspectives [18,19]. A single survey instrument was used, which included questions collecting both quantitative and qualitative information (see Table A1 in the Appendix A). Respondents were queried about their dietary habits, experiences with alternative proteins, and their perceptions and willingness to consume these products in the future. The survey also sought to understand their concerns about the quality, safety, health risk and nutritional value of alternative proteins, as well as their general attitudes toward incorporating these proteins into their diets. NVivo 14, Windows version, developed by Lumivero for qualitative data analysis [20], was used to organise, code survey data, identify patterns, analyse, and visualise the participants’ responses. The software also allows to calculate percentages of responses containing a particular coded category. An ethics permit for the involvement of human subjects in this study was obtained from Curtin University Human Research Ethics Committee (HRE2022-0041), and the survey was conducted online in 2024, using the open-source online survey tool LimeSurvey 6.0 [21]. Participants were randomly recruited from a database of people who had signed up to voluntarily participate in surveys. All participants acknowledged electronically (ticked box) an informed consent statement in order to participate in the study survey, and their responses were to be reported anonymously.

### Sample Description

A total of 520 individuals across different age groups, ranging from 18 to 64 years old, participated in the survey. We approached 1000 randomly selected people, and 520 completed the survey, resulting in a 52% survey response rate, which is higher than the average survey response rate of 33% [22]. The demographic characteristics of the respondents are presented in Table 1. This sample provides the foundation for understanding consumer perceptions and behaviours regarding alternative proteins.

The survey participants’ sample was evenly distributed between males (49.6%) and females (50.4%), ensuring balanced insights from both genders. This balanced gender split enhances the generalisability of the findings across the two main consumer groups. The respondents are evenly distributed across the age cohorts between 1960 and 2005, with each 10-year cohort comprising roughly 20% of the sample. This allows for comparisons across generations, including any generational differences in attitudes and behaviours toward alternative proteins.

A significant portion of the respondents hold a bachelor’s degree (38.1%), followed by TAFE/college education (21.3%) and high school education (18.4%). Advanced degrees (master’s 15.2% and PhD 2.5%) were less common. The educational profile of the respondents closely aligns with the latest Australian Bureau of Statistics (ABS) census data from 2021, confirming that the sample is statistically representative. In 2021, 5.5 million people (30.7% of the population) reported having a bachelor’s degree or higher, and 4 million people (21.3% of the population) reported having a Certificate I to IV, which includes TAFE and vocational education [23]. This alignment suggests that the study reflects broader socioeconomic and educational trends, particularly the link between higher education, sustainability awareness and health consciousness, which may potentially influence dietary choices and consumer behaviour. Most respondents were either married with children (38.8%) or single (36.9%). A smaller proportion were those in de facto relationships (13.5%) or married without children (9.6%), and divorced individuals represented only 1.2%. Family status could influence food purchasing, preparation and dietary decisions, as married individuals with children might prioritise family-friendly and accessible food options, whereas single individuals might experiment more with alternative proteins.

A substantial portion of the participants reported themselves as daily meat eaters (45.2%), confirming Australia’s strong meat culture. A further 20.4% fall into the frequent meat consumption (4–5 times per week) category, underscoring Australia’s meat-heavy diet. Flexitarian and occasional meat consumption was shared by 30.2% of the survey respondents, who claimed they consumed meat less than four times per week, reflecting a growing segment of people reducing their meat intake. Vegetarians and vegans combined represented only 4.2% of the respondents. Among them, 2.9% identified themselves as vegetarian and 1.3% as vegan. While meat remains dominant, there is some evidence toward reduced meat consumption, which aligns with the increasing interest in alternative proteins or preferences for plant whole-food options [1].

Among the participating respondents, males were more likely to be daily meat eaters (45%) compared to females (35.9%). Conversely, females were more likely to identify as flexitarians (34.3%) or vegetarians (4.6%) than males (14.3% and 1.2%, respectively). This indicates potential gender-based differences in attitudes toward meat consumption, with women possibly being more open to reducing meat intake and adopting alternative foods.

## 3. Results

### 3.1. Experience with Alternative Proteins

In Australia, all main groups of alternative proteins are readily available, except for cultured meat. There is a large variety of plant-based milk, meats and products containing algae, while insect-based proteins are sold in specialised shops and in the form of powder in some of the major supermarkets. Cultured meat is currently sold only in specialty restaurants in Singapore, the first country to approve cultivated meat [24]. Table 2 shows the survey participants’ experience with alternative proteins, while Table 3 indicates their intentions to continue consuming these foods in the future.

The survey results indicated a positive perception of plant-based milk and algae alternatives, which stand out for their relatively high trial and future consumption rates. Plant-based milk had the highest trial rate (74.8%) among all alternative proteins, with 21.9% of participants intending to consume it regularly and an additional 23.1% occasionally. Among the variety of plant-based milk available on the Australian market, soy milk comes the closest in nutritional profile to cow’s milk [25]. Not all plant-based milks contain a nutritionally significant amount of protein, although some may be fortified, but for the purpose of this study, we did not distinguish between different brands and varieties. Algae-based products followed closely in popularity, with 91.9% of participants having tried them and 9.4% willing to consume them regularly. Some algae, such as chlorella, spirulina, and other blue-green and green algae, contain high levels of protein [26], while others are lower in protein. The protein content in algae also depends on the processing method [27], but again for the purpose of this study, we did not distinguish between different species. It is likely that the established presence of algae in certain cuisines, such as sushi and seaweed salad, contributes to their acceptance, although these products may not be specifically eaten as a protein source. The perceived health benefits, environmental sustainability, established use in familiar products and less cultural resistance may explain their popularity.

In contrast, plant-based meat received a neutral to low reception among the survey participants, with their intention for future consumption remaining relatively low. While 57.3% had tried plant-based meat, only 1.5% intended to consume it regularly and 17.7% occasionally, with the majority (80.8%) unwilling to include it in their diet. Concerns about health risks, taste, texture and processing likely contribute to this hesitation. Additionally, cultural associations with masculinity may deter male consumers from occasional to regular consumption of these products [28]. Perceptions about plant-based meat being ultra-processed may similarly deter consumption [14].

Novel protein sources such as insects and cultured meat faced significant resistance and received low to negligible reception. Only 21.5% of respondents had tried insects, and none intended to consume them regularly, while 89.8% stated they would never eat them. Similarly, cultured meat had the lowest trial rate (0.2% and consumed overseas, as this option is not available in Australia) and was rejected by 97.3% of participants for future consumption. This strong aversion is likely due to food neophobia, cultural taboos, unfamiliarity, more restricted availability, and uncertainty regarding their safety and ethical implications. The reluctance to accept cultured meat could be due to lack of availability (it is currently available only in Singapore), high costs, scepticism about safety and limited public awareness.

As sensory attributes, taste and texture are pivotal for any food product’s success. Poor experiences and perceptions of these qualities inhibit adoption. A significant number of respondents, 53.6% (n = 279), who have tried alternative proteins expressed dissatisfaction with their taste and texture. Previous experiences, particularly with the taste and texture of plant-based burgers, which participants claimed did not meet consumer expectations compared to conventional meat, contributed to this dissatisfaction. Some described plant-based proteins being overly fatty, greasy and leaving an unpleasant aftertaste.

*“I tried a plant-based burger at a friend’s place, and it didn’t meet my expectations. It was too greasy and had an unpleasant aftertaste.”*

(Male, born 1997)

Dissatisfaction with taste and texture acts as a barrier to adoption, as consumers often compare these alternatives directly with the sensory qualities of traditional meat. The excerpt below is indicative of many similar comments:

*“My issue lies with the taste. It’s nothing like that of conventional meat. I think that the taste could not be mastered with any of the alternatives.”*

(Female, born 1998)

Such negative experiences were particularly vivid amongst younger (Generation Z) participants, with 21.7% (n = 113) of them being dissatisfied. The qualitative data presented in Table A2 in Appendix A capture some of the expressed views. Young people, who are increasingly exhibiting independent dietary preferences, are likely to determine the future of any novel foods.

Some of the survey respondents, 10.7% (n = 56), across different age groups found the alternative proteins unappetising and explicitly stated they would not consume them again or give them to their children or relatives. Table 4 captures some of the opinions shared.

One of the participants had a very clear message for the industry that its products need to be improved.

*“Trying these alternatives does not mean I will consume them regularly. People are often curious to explore new stuff, so do I … I think they are nutritionally not compatible to meat and not nice. If their producers want to have a slight chance their product to be out there and met with interest from the consumers, in the future, they need to continue to improve their taste, texture, appearance, and especially nutritional quality.”*

(Male, born 1999)

The situation with plant-based milk, however, was different, with much wider acceptance (see Table 3) and recognition of their benefits (see Table 5). Plant-based milks were seen as a potentially good option, particularly for those with lactose intolerance. Some participants also expressed a tentative openness to considering insects as an alternative protein source, although there was a widespread lack of familiarity with these options.

Across generations, the data reveal a clear generational trend in plant-based milk adoption, with Gen Z leading in both trial and future consumption intent. A significant 33.3% of Gen Z respondents have tried the product, the highest among all age groups, indicating their openness to experimentation and new food choices, likely influenced by digital engagement and social trends. In contrast, millennials (21.7%), Gen X (12.3%), and baby boomers (7.5%) show a gradual decline in plant-based milk product trial rates, suggesting that older generations are more resistant to adopting new dietary habits (see Table 6).

Plant-based milk’s future consumption intent follows a similar pattern, with 34.0% of Gen Z respondents expressing interest in future consumption, making them a key target market. Millennials also show potential at 22.9%, whereas Gen X (23.9%) and baby boomers (19.2%) exhibit lower levels of interest. Despite high trial rates, regular consumption remains relatively low across all generations, with only 10.4% of Gen Z and 7.1% of millennials consuming the product regularly (see Table 6). This suggests that while younger consumers are open to trying new foods, they may not yet consider this product a staple in their diet.

Resistance to trying plant-based milk is most pronounced among older generations, with 11.5% of Gen X and 11.7% of baby boomers having never tried it, compared to just 0.8% of Gen Z and 1.2% of millennials. Moreover, 19.8% of Gen X and 14.2% of baby boomers state they will never consume it (see Table 6), highlighting entrenched food preferences and potential scepticism toward novel food trends. Consumption of plant-based milk clearly lacks familiarity and habitual appeal or is considered more of an experimental or niche choice rather than a staple. These insights suggest that while Gen Z and millennials present strong growth opportunities, marketing strategies for Gen X and baby boomers must focus on education, health benefits and familiarity-building to reduce resistance. Older generations’ resistance highlights the need for targeted messaging that addresses concerns and promotes benefits that resonate with their values (e.g., health, tradition or sustainability).

On the other hand, there were not any pronounced trends according to educational level when it comes to trying plant-based milk (see Table 7). Nevertheless, with increased achieved educational levels, there seems to be higher determination not to consume plant-based milk in the future, although the sample is relatively small. Similarly, the share of participants reporting intention for occasional future consumption also decreases with higher levels of education achieved.

Overall, the data suggested that younger generations are shaping the future of food choices, with Gen Z leading the way in openness to new products and trends. However, low regular consumption across all groups presents an opportunity to enhance product appeal, whether through taste, convenience or perceived health benefits. Addressing generational differences in adoption and resistance will be crucial for expanding a product’s market penetration.

The sections to follow are based mainly on the qualitative information provided by the participants. They also indicate serious levels of concern and resentment among Australian consumers. Excerpts from the survey texts are quoted verbatim to give voice to the stated concerns and convey the feelings of the participants in the study.

### 3.2. Health-Related Issues

Concerns about safety, alternative proteins being ultra-processed foods and potential health risks emerged as significant barriers to adopting them. Participants expressed uneasiness about the production methods, lack of regulatory oversight and long-term implications of consuming these novel foods.

#### 3.2.1. Food Safety and Regulation

The survey findings revealed that there is a significant emphasis on the need for stringent regulation and traceability of ingredients in alternative proteins. Participants frequently (one in every five of the survey respondents or 20%, n = 104) mentioned food safety and quality control as issues of concern.

*“A bit of sceptical about the alternative proteins’ quality. I am wondering whether there is any regulatory body … that tests the quality and safety of alternative proteins before they are introduced to the market. I think this is important for consumers.”*

(Male, born 1995)

A recurring concern (14%, n = 74 of all participants), particularly among Generation Z participants (one in every three or 33%, n = 59), was the health credentials of these novel foods and the safety risks associated with them. This highlights the necessity for robust regulatory frameworks to ensure the safety and quality of alternative proteins.

*“I think they pose food safety risks to the consumers.”*

(Female, born 1999)

Younger participants expressed doubts about the health benefits of these products, citing issues such as heavy processing and the inclusion of unfamiliar ingredients to mimic the taste of meat.

*“Look, my generation is super mindful of what we eat. But let’s be real—alternative proteins? They’re not exactly the ‘smart’ choice everyone makes them out to be. The problem isn’t just that they’re ultra-processed; it’s that we don’t actually know how safe they are in the long run. A lot of these products are packed with artificial ingredients just to mimic the taste of meat, but no one’s talking enough about what that means for our health years down the line. Why should we blindly trust something that hasn’t been properly tested? It feels like we’re being told it’s the future of food, but honestly, I’m not convinced.”*

(Female, born 2003)

This statement is not held alone amongst younger consumers. It reflects a deep scepticism toward alternative proteins, highlighting concerns about their high level of processing and the unknown long-term health effects of their ingredients. The frustration is evident, particularly in the way they question the lack of thorough testing and transparency in the industry. This perspective aligns with a broader Gen Z mindset, one that values authenticity, transparency and evidence-based decision-making when it comes to food choices. While alternative proteins are often marketed as the future of sustainable eating, many young consumers remain unconvinced, fearing that these innovations prioritise profit and convenience over genuine health benefits. Ultimately, this scepticism underscores the need for more research, clearer labelling, and open discussions about the safety and long-term impact of novel food technologies. Younger Gen Z participants’ reluctance to embrace alternative proteins due to perceived health risks and processing concerns suggests that producers need to address these issues to gain acceptance among this demographic. For these participants, understanding how plant-based meats and other alternatives are made is essential in determining whether they align with their values and dietary choices. This generation’s influence on future food trends makes their concerns especially pertinent.

A proportion of older participants (7.1%, n = 37), representatives of Gen X and baby boomers explicitly expressed doubts about the transparency of ingredients and placed a strong emphasis on food safety and the adequacy of regulatory frameworks.

*“Novel foods need to be developed up to the needed food safety and quality standards that properly regulate the use of ingredients, processing aids, colourings, additives. Otherwise, I am uncertain about them.”*

(Female, born 1974)

Participants also raised concerns about ingredient traceability.

*“Everything about this food is novel to me. I believe we need to know more about how plant-based meats and other meat imitations are made. We need to be able to trace the ingredients used in their development and monitor them to ensure they are safe options for us to consume if we must consume them.”*

(Male, born 1968)

These statements reflect a cautious approach toward novel foods, particularly among participants who were traditionally raised with meat. Their concerns are not about outright rejecting alternative proteins but ensuring these products meet the same rigorous food safety and quality standards as conventional meat. They emphasise the need for clear regulatory frameworks and strict oversight, advocating for novel foods to be held to the same standards as all other food products. Additionally, their focus on ingredient traceability underscores a desire for informed decision-making, ensuring that if they choose to consume meat alternatives, these products are not only safe but also trustworthy. Their perspective suggests that greater transparency and clearer communication from food producers and regulators could help bridge the gap between curiosity and cautious acceptance. To overcome these barriers, it is crucial for producers to enhance the safety, transparency and health credentials of their products.

Contamination was another important issue of concern for 13.2% (n = 69) of the respondents who highlighted issues related to both manufacturing process and storage (see Table 8). Participants worried about microbiological risks from improper handling in supermarkets, as well as the potential for chemical, microbial, physical or allergenic contamination in alternative proteins. These concerns emphasise the need for stricter safety protocols, clearer labelling, and better storage practices to enhance consumer confidence in alternative proteins.

Not all participants viewed alternative proteins negatively. Some respondents (8.8%, n = 46) expressed trust in their safety, particularly in products perceived as well-regulated (see Table 9). These responses reflect a general trust in the regulation and safety of alternative proteins. Additionally, 16.7% (n = 87) of the survey participants trusted selectively only certain alternative proteins, particularly seaweed and soy milk, with which they were familiar and perceived as safe. It appears that people’s opinions are quite divided, and more reassurance is required to build consumer trust and familiarity.

#### 3.2.2. Artificial Ingredients and Processing

The highly processed nature of alternative proteins was criticised by many participants (18.6%, n = 97). This was making them less desirable. Furthermore, they felt that additives used to enhance taste and texture made these products less appealing (see Table 10). Particular concern was raised (12.5%, n = 65) for the use of additives, such as carrageenan, methylcellulose and artificial colouring, which were associated with health risks, including cardiovascular diseases and mental health disorders. This attitude in the mind of consumers ties into broader apprehensions about the potential long-term health impacts of consuming heavily processed foods. Many (14%, n = 73) emphasised a strong preference for natural, minimally processed, whole plant foods over heavily processed alternatives, viewing the artificial nature of these products as harmful and incompatible with their dietary values. There was notable distrust and fear that reliance on artificial ingredients could worsen public health rather than address food security issues.

Specifically, concerns about the potential use of genetically modified organisms (GMOs) in products like soy were expressed by many respondents (11.3%, n = 59). This was making the participants cautious and reluctant to consume the new alternative plant-based proteins (see Table 11).

The majority of the respondents (51.3%, n = 267) commented on the long list of ingredients and expressed a strong need for greater transparency from producers regarding what they are, their sourcing, additives used and the manufacturing processes involved. Many viewed these additives as overly processed and unhealthy. Some saw this as a primary issue of concern, suggesting that while these additives may be necessary to enhance the texture and taste of the plant-based analogues, they are not necessarily healthy or safe for consumption (see Table 12). This concern contributes to the overall distrust of these food products. It also implies a suspicion that the complexity and artificial nature of these additives could potentially be harmful or at least not as beneficial as natural meat.

*“I am concerned because I don’t trust the producers. They always try to show they are focused on using natural ingredients, like good protein from peas, soy, mung beans, and etc., for their meats, but they do not explain the way they used these supposedly healthy plant ingredients, which is a must nowadays.”*

(Female, born 1977)

Respondents focused their comments on the quality of plant-based meat and milk alternatives, with almond milk generally viewed as less concerning. However, there was noticeable unfamiliarity with other options, such as insects, lab-grown meat and lab (or synthetic) milk. Overall, the participants showed a clear resistance to adopting alternative proteins, expressing a strong preference for natural and traditional food choices.

Continual improvement of taste and texture to provide a comparable or superior experience to traditional meat or dairy products was identified by the respondents as crucial. Currently seen as nutritionally inferior to livestock-sourced foods and less enjoyable in terms of taste, alternative proteins were seen as less desirable. Those who have already tried them were not committed to continuing to consume them in the future.

#### 3.2.3. Allergens and Health Risks

Allergies triggered by the ingredients contained in the alternative proteins were highlighted as a health risk, particularly when people have not been exposed to such substances in the past or are unfamiliar with the contents list (see Table 13). Many respondents (13.3%, n = 69) were particularly concerned about allergies caused by insects as well as cross-contamination with allergens during the manufacturing process. Female participants appeared to be more aware of this issue. Allergies can be a serious health threat, and in addition to a detailed list of ingredients, consumers should be advised to taste the new products with caution.

Many of the survey respondents (11.2%, n = 58) raised potential health risks associated with the alternative proteins, such as carcinogenic properties when cooked at high temperatures, cardiometabolic and human microbiome issues (see Table 14). Addressing such concerns requires transparent ingredient labelling, reduced sodium levels and independent health risk assessments, which can help mitigate such attitudes.

For many participants, eating alternative proteins was not a healthier option than other traditional products, such as lean meat or vegetables and fruit (see Table 15). Some also questioned the aspiration to imitate the taste and texture of meat.

### 3.3. Nutritional Value

Participants exhibited a generally cautious attitude toward the nutritional value of alternative proteins, expressing scepticism and concerns about their ability to replicate the nutrient profile of traditional meat. Concerns about nutrient deficiency and unhealthy nature made alternative proteins acceptable for some only for occasional consumption.

#### 3.3.1. Nutrient Deficiency

Concerns about micro- and macronutrients and dietary fibre content in artificial meats were expressed as significant by 21.7% (n = 113) of the respondents (see Table 16). Respondents were unsure about comparisons with livestock meat and milk, expressing doubts about protein content and the presence of essential nutrients such as calcium, iodine, vitamin B12, iron, zinc and omega-3 fatty acids. These deficiencies were attributed to the absence of naturally occurring nutrients in the plant-based alternatives and the fact that they are often artificially supplemented. Some participants doubted the effectiveness and bioavailability of these added nutrients, perceiving them as inferior to those naturally present in animal-based products.

Some (7.3% n = 38) suggested that whole plant foods, algae and insects might offer better nutritional profiles but are hindered by negative perceptions or potential allergens. Overall, the consensus was that while alternatives might provide certain dietary fibres and vitamins, they fall short of matching the comprehensive nutrient profile of traditional meat and dairy products, leading to concerns about their long-term health implications. The concerns expressed by the respondents point to a need for clearer nutritional labelling and education to reassure consumers about the nutritional benefits of alternative proteins.

A participant was critical of plant-based protein alternatives, arguing that while they are often marketed as promoting superior health outcomes, they are nutritionally inadequate despite the additives included by producers.

*“Plant foods’ category is associated with superior health outcomes, but in fact, they are nutritionally poor despite what their producers have added into their ingredients, some vitamins and minerals. The only good option is perhaps plant-based milks and perhaps the insects.”*

(Female, born 1968)

Amongst the alternative proteins present on the market, plant-based milk was perceived more favourable. The trust in the nutritional benefits of plant-based milk compared to plant-based meats was noticeable among a quarter of the participants (24%, n = 125). These respondents are satisfied with the products they consume currently and planned to continue consuming plant-based milk.

*“I tried some of the alternative proteins’ products like fake meats once out of curiosity. I wasn’t satisfied at all. I think the claims producers are making for these products are not substantiated, especially with the taste provided, their look, behaviour when cooking. Perhaps oat milk is okay with the taste that is currently providing, but the rest are greasy, salty, and tasteless non-sense, especially the vegan burgers.”*

(Female, born 2000)

#### 3.3.2. High Sodium Content

High sodium content (see Table 17) was also highlighted by many participants (21%, n = 110) as an undesirable characteristic of the new foods in an attempt to improve their taste qualities. It was associated with non-communicable diseases, such as cardiovascular disease and high blood pressure. Again, it is important to provide accurate information on the packages to inform the consumer about the amount of salt in the alternative proteins.

#### 3.3.3. Occasional Consumption

Around 15% (n = 78) of the respondents were of the opinion that alternative proteins can be acceptable for occasional consumption (see Table 18). They compared them to other discretionary foods, such as fast food or chocolate, which are not healthy but are still commonly consumed. Just like fast food, plant-based meat alternatives undergo significant processing to achieve desired textures, flavours and nutritional profiles, often involving artificial ingredients, preservatives and flavour enhancers. This level of processing can strip away some of the natural nutrients found in whole foods and introduce substances that some consumers might perceive as less healthy. As a result, while these alternatives offer a more sustainable and ethical choice compared to animal products, their nutritional benefits are sometimes viewed with scepticism, placing them in the same category as other convenience foods that are not consumed for their health benefits but for their taste, convenience and enjoyment.

Some respondents shared their intention to not disregard the existence of alternative proteins and try these options despite their initial disbelief in the nutritional quality and without high expectations about these products.

*“Plant-based and growing food from animal cells in a lab are seen as potential solutions to food security, but if these are not nutritionally sane, this means food safety and quality of these products is not there.”*

(Male, born 1958)

### 3.4. Consumer Attitudes

Ultimately, consumer attitudes will decide the future of alternative proteins. The survey participants emphasised the importance of transparency about the production process, ingredients and nutritional value of the new food products, but they also questioned how such foodstuff would fit within the Australian landscape, where meat consumption is intertwined with perceptions about strength and masculinity. Furthermore, there were some generational differences between the Australian consumers. All these are discussed below.

#### 3.4.1. Transparency for Informed Decision

Many participants spoke loudly (18%, n = 94) about the need for transparency regarding the ingredients and production processes for alternative proteins to be able to make informed consumer choices. They objected to the marketing of these products without a clear understanding of the potential nutritional and health implications. Insufficient information also gave rise to scepticism and concerns, as indicated earlier (see Table 19).

There were a few voices (4.4%, n = 23) that indicated a level of trust in the manufacturers.

*“If there are any nutritional or other health-related issues, I think the manufacturers and producers can address them promptly.”*

(Male, born 1965)

They seemed to trust that any potential problems could be promptly resolved by the companies producing these products. This perspective indicates a level of faith in the regulatory processes and the responsiveness of the industry to ensure the safety and nutritional adequacy of alternative proteins.

However, the majority of Australian consumers want transparency around the food product origin, processing and ingredients together with clear, accurate, easily accessible and comprehensive nutritional information. This could help educate consumers about alternative proteins, fostering a better understanding of their benefits and potential drawbacks. By being transparent, producers can build trust in consumers, who otherwise may remain sceptical about the quality and safety of these products. Such an approach can lead to better-informed choices and increased acceptance of alternative proteins.

While there was widespread scepticism about plant-based meat substitutes, there appeared to be wider acceptance of seaweed, especially in sushi, and plant-based milk, such as soy, oat and almond, as safe and acceptable alternatives. This view highlights a preference for plant-based options perceived as more natural and nutritionally reliable, contrasting with the distrust toward more processed meat substitutes.

#### 3.4.2. Masculinity

A small number of male respondents, 3.1% (n = 16), and one female participant expressed a view about alternative proteins being not masculine and, therefore, unwanted and unsafe for males to consume. Despite the small number of people raising this socio-cultural concern, it is significant because it emerged spontaneously without participants being specifically asked about the link between alternative proteins and masculinity. The association between meat consumption and masculine identity is deeply ingrained in the Australian psyche [29], and such a cultural and identity-related factor needs to be taken into account.

The respondents’ opinions predominantly express strong opposition to alternative proteins, primarily rooted in traditional notions of masculinity and the cultural association of meat with male identity, a characteristic of male consumers in Australia and internationally. They voiced strong preferences for traditional meat over alternative proteins, which they found more masculine and with the right sensory appeal of “juicy”, “bloody”, “more manly”, “nutritiously wiser” and for “real man” compared with the meatless alternatives. Some of these participants dismissed other alternative proteins based on insects and seaweed, as improper, not nutritious and unsafe for males. Concerns extend beyond taste and texture, with some believing that consuming alternative proteins could undermine their masculine image or affect testosterone levels (see Table 20).

The female respondent believes that alternative proteins are reinforcing gender stereotypes by primarily targeting women and not addressing the perceptions that make these products unappealing to many males.

*“I think the new alternative proteins are targeting more females than males. The focus should be on ensuring these alternatives are safe, nutritious, and appealing to a wide audience, but they are also reinforcing gender stereotypes, as many males will not even touch them as they are perceived unmasculine.”*

(Female, born 1986)

The presented perspectives reflect a deep-rooted belief in the superiority of livestock meat among male consumers. This belief is influenced by cultural and personal notions of masculinity, which interfere with the acceptance and consumption of alternative proteins. Masculinity is a point of concern and a barrier to the future consumption of alternative proteins. These sentiments indicate major barriers, particularly for men who associate masculinity with meat consumption. The presented views underscore the need for these alternatives to be marketed as safe, nutritious and appealing to everyone without reinforcing existing gender stereotypes. This is why it is important to positively promote meat alternatives [30] so men do not feel pressured to consume something that compromises their masculinity traits [28]. For the future of alternative proteins, addressing these deeply ingrained cultural and identity-related perceptions will be crucial, potentially through targeted marketing strategies and educational campaigns that redefine masculinity in the context of alternative food choices. Responding to these concerns may require nuanced approaches that respect traditional views while encouraging broader acceptance of sustainable and ethical food choices.

#### 3.4.3. Generational Attitudes

There were some generational differences in consumer attitudes toward alternative proteins. General scepticism, reluctance and worry were prevalent among adult Generation Z (1995–2006) and people born 1960–1969—considered roughly parents of Generation Z (see Table 21). By comparison, respondents born between 1980–1989 were open to consuming alternative proteins, and 8.3% (n = 43) of them were not aware of any associated specific problems. They generally trusted these products to be safe and regulated, with confidence that any potential nutritional issues would be addressed by the producers.

### 3.5. Environmental and Ethical Considerations

A major advantage of the alternative proteins is their lower environmental footprint; however, this is not enough for Australian consumers. Although some participants (19%, n = 99) expressed a desire to be more environmentally friendly in their food choices, doubts about the nutritional and health impacts of the alternative proteins prevailed (see Table 22). Communicating the environmental benefits of alternative proteins alongside robust nutritional data could help align ethical motivations with health expectations.

## 4. Discussion

Alternative proteins are finding their way onto supermarket shelves and on restaurant menus. Research on consumer attitudes toward them is catching up in Australia and around the world. Dietary changes, however, are slow and difficult to shift [31], and Australia has a large proportion of people unwilling to reduce their meat consumption [15]. It is important to stress that consumers expect alternative proteins to be able to match or exceed the organoleptic quantities of the respective animal-based products, including meat, and also to be similarly priced. Despite significant efforts to achieve parity with animal-sourced foods, currently, there are numerous challenges with the flavour, texture, taste, and other physicochemical and functional attributes of alternative proteins, as well as with their nutritional profiles, safety, and production costs [32,33].

Similarly to Onwezen et al. [34], this study confirmed that it is highly relevant to differentiate between different alternative proteins. Plant-based milks appear to be widely acceptable, with 45% of the participant Australians happy to consume them on a regular basis or occasionally. This is partially triggered by lactose intolerance prevalent in people from East Asian decent [35], with two-thirds of Australian Asian population having problems digesting lactose [36]. Dietitians estimate that one in four (namely 27%) of Australian households have members who are lactose intolerant [37]. For these consumers, plant-based milk represents a good substitute, and many participants stressed these benefits. A further benefit is the potential sustainability advantages of plant-based milk in terms of the use of land, water, fertilisers and generation of lower greenhouse gas emissions [38]. The survey questionnaire, however, did not capture nuances in consumer attitudes toward different plant-based milks, which can be explored in future research.

Familiarity with the alternative proteins [39] played a big role in the relatively high acceptance of algae, with more than half (50.6%) of the participants prepared to consume them on a regular or occasional basis. Sushi, seaweed biscuits and seaweed-based ingredients used in popular snacks are a driving force behind consumers’ familiarity and trust in these alternative proteins [40]. The study respondents also emphasised the naturalness of algae, which makes them acceptable. On the other hand, despite insects also being natural, a very few (10%) were prepared to eat them and only occasionally. Those who have tried insects (22%) may have done this during their travel overseas, although a range of products with insects are available in Australia in specialty shops. Potential allergies and aversion were the main reasons for rejecting this type of alternative proteins. In this respect, the Australian study participants do not differ from consumers in other Western countries, such as USA [41] or Italy [42]; however, food neophobia [43,44]) did not explicitly emerge as a contributing factor.

The study indicated that uncertainty surrounding the long-term health and safety implications of regular intake of alternative proteins remains a major force preventing consumption and a significant barrier to acceptance [45,46]. This scepticism is not necessarily about rejecting innovation but rather a cautious response to the unknown. Without clear, evidence-based assurances from food regulators and producers, concerns about contamination, ingredient safety and potential health risks overshadow even positive sensory experiences. As a result, many consumers hesitate to embrace these products fully, reinforcing the need for greater transparency, rigorous testing and clearer communication to build trust in novel food alternatives.

An interesting, but not unexpected, finding from this study is the similarity of consumer attitudes of people following different diets. For example, flexitarians were not more likely to be interested in consuming the alternative proteins; neither were occasional meat eaters, vegetarians, nor vegans. As the share of Australians who regularly consume livestock-sourced products is very high (86.2% in our sample), it is best to target people in the mainstream by providing guidance on the benefits of consuming plant protein while reducing reliance on animal sources. Plant-based meat alternatives can play a role in this, particularly if they are made from sources, such as legumes, which can “emulate the or sensory properties and functionality of the foods they are intended to replace” ([47], p. 392). Familiarity with products, such as lentils or tofu, can facilitate consumer acceptance. Similar to Messina et al. [47], a good strategy from a health, safety, environmental and ethical point of view is to encourage an increase in the intake of legumes consumed in a traditional manner combined with some alternative proteins as a way to transition to a plant-predominant diet. This would align well with the consumers (15% of the participants) who see the alternative proteins as acceptable for occasional consumption.

The current quality of the alternative proteins leaves a lot to be desired. Many respondents (53.6%) were dissatisfied with the taste, texture and aftertaste of these products, particularly the plant-based burgers, describing them as “fatty”, “greasy” and “unappealing”. A 2023 study of plant-based beverages and plant-based cheeses found that increased aftertaste led to decreased liking of these products [48]. The lack of standardised methods for testing sensory experiences with the new alternative proteins is challenging, both for producers and consumers [49]. Negative experiences have led some Australian participants to firmly decide against consuming these alternatives in the future, especially when it comes to feeding their children. Taste quality highlights a significant barrier to acceptance and regular consumption of plant-based meat products.

Even with some interest in trying alternative proteins, many respondents have significant reservations about their nutritional value, safety, ingredients, taste, processing and potential health risks. These concerns highlight the need for clearer communication from producers, better regulatory oversight, and further research to address these issues and build consumer trust [45]. Communicating the environmental benefits alongside robust nutritional data could help align ethical motivations with health expectations.

Our study revealed some generational differences among consumers, with Generation Z and their parent generation being less inclined to consume alternative proteins. Although previous research indicates that women may have a better predisposition toward alternative proteins [50], there were no significant differences in opinion between the male and female participants in our study. Nevertheless, the cultural issue about which food is perceived as appropriate for men was raised. Alternative proteins were seen as impacting perceptions of masculinity. This confirms the findings from earlier studies in Australia [28] that men are very sensitive as to how plant-based alternative proteins would impact their manly image.

As the same concerns about adequate protein intake and performance persist and are applied to any plant-based foods, including fruit, vegetables, nuts, legumes and roots, as well as fungi, in addition to information about the benefits of these proteins, it is important to also use male role models of top-performing athletes who eat a plant-rich diet. Athletes such as Novak Djokovic and Lewis Hamilton, as well as the Australian Nick Kyrgios and Kane Richardson, have demonstrated that a plant-rich diet can support peak physical performance [51]. They are examples of male athletes who have embraced plant-based nutrition, challenging traditional assumptions that animal protein is essential for athletic success.

The highly processed nature of alternative proteins combined with long lists of ingredients were common concerns for the study participants, with many perceiving these products as artificial and therefore less desirable. Potential allergens, GMOs and contamination were similar concerns raised, intermixed with a lack of trust in the integrity of food manufacturers. This ties into broader fears about the potential long-term health impacts of consuming heavily processed foods. Some respondents were worried about specific health risks, such as carcinogenic properties and impacts on the microbiome, which suggests a need for more research and transparent communication about the safety of these products. The findings from our study support the challenges faced by the US alternative proteins industry in delivering healthy and sustainable diets [52], namely a confusing marketing landscape with scepticism among consumers, insufficient information through product labelling and education, as well as inadequate policies and regulations.

A further research direction could be to investigate how successful the food industry has been in influencing people’s perceptions about nutritional equivalency between traditional and alternative proteins. The study also did not explicitly ask questions about affordability and accessibility of alternative proteins because this varies vastly, and also, the costs of new products tend to start higher, and wider adoption stimulates economic efficiencies and technological innovation. These aspects can also be explored in further research. Animal welfare and how this impacts consumer food choices and adoption of alternative proteins can also be investigated, including from a generational perspective.

## 5. Conclusions

In conclusion, the findings of this study confirm that Australian consumers are not yet ready to fully embrace the majority of alternative proteins. They indicated a diverse range of concerns, highlighting both personal preferences and broader health and safety issues. While environmental and ethical considerations drive curiosity, deep-seated concerns about nutritional adequacy, ingredient transparency and food safety remain major barriers. Many respondents expressed scepticism about the nutritional value and the health benefits of alternative proteins, fearing that they may lack essential nutrients while being overly processed and loaded with additives to replicate the taste and texture of traditional meat. This uncertainty fuels hesitation, even among those who are open to reducing meat consumption.

Consumer concerns extend beyond personal preferences to include broader issues such as food safety, regulatory oversight and the sensory qualities of these products. Negative experiences, such as unpleasant taste, texture or aftertaste, significantly impact consumers’ opinions and reinforce resistance. It suggests that product quality and formulation must improve significantly before widespread acceptance can be achieved. Addressing these barriers requires a multi-pronged approach, which combines stronger regulations, transparent and detailed labelling, and clearer communication about the nutritional content and safety of alternative proteins. Without such efforts, even environmentally conscious consumers will remain hesitant. Balancing ethical and environmental messaging with robust nutritional information can help build a more comprehensive and trustworthy narrative.

Importantly, alternative proteins should not be positioned as a direct substitute for traditional meat but rather as an option and part of a broader shift toward a more plant-forward diet. While certain products, such as plant-based milk and algae, have gained considerable acceptance, others, like plant-based burgers, are viewed more as an occasional option rather than a staple. Some options, like cultured meat, are considered not feasible at all. To gain a lasting foothold in the market, alternative proteins must move beyond novelty status by proving their nutritional benefits, improving sensory appeal and fostering greater consumer trust. Ultimately, given the complex landscape of available products and consumer concerns, alternative proteins should be seen as complementary to, rather than replacing, traditional meat proteins. This approach can help facilitate a transition toward a more balanced and sustainable food future.

## Figures and Tables

**Table 1:** Respondents’ demographic characteristics.

Demographics	Male	Female	Total
	49.6% (n = 258)	50.4% (n = 262)	100% (n = 520)
Year born			
2000–2005	19.6% (n = 102)		
1990–1999	20.2% (n = 105)		
1980–1989	20% (n = 104)		
1970–1979	20.2% (n = 105)		
1960–1969	18.8% (n = 98)		
1950–1959	1.2% (n = 6)		
Educational level			
Year 10	4.4% (n = 23)		
High school	18.4% (n = 96)		
Technical and Further Education (TAFE)/College	21.3% (n = 111)		
Bachelor	38.1% (n = 198)		
Master	15.2% (n = 79)		
PhD	2.5% (n = 13)		
Family status			
Single	36.9% (n = 192)		
Married with kids	38.8% (n = 202)		
Married without kids	9.6% (n = 50)		
De facto	13.5% (n = 70)		
Divorced	1.2% (n = 6)		
**Dietary behaviours**	**Male**	**Female**	**Total**
Daily meat eater	45.0% (n = 116)	35.9% (n = 94)	45.2% (n = 235)
4–5 times meat a week	26.4% (n = 68)	14.5% (n = 38)	20.4% (n = 106)
Flexitarian 2–3 times meat a week	14.3% (n = 37)	34.3% (n = 80)	17.7% (n = 117)
Occasional 1–2 times meat a week	12.4% (n = 32)	12.6% (n = 33)	12.5% (n = 65)
Vegetarian	1.2% (n = 3)	4.6% (n = 12)	2.9% (n = 15)
Vegan	0.8% (n = 2)	1.9% (n = 5)	1.3% (n = 7)
Total	100% (n = 258)	100% (n = 262)	100% (n = 520)

**Table 2:** Have tried alternative proteins.

Alternatives Types	Tried	Never Tried
Plant-based meat	57.3% (n = 298)	42.7% (n = 222)
Plant-based milk	74.8% (n = 389)	25.2% (n = 131)
Insects	21.5% (n = 112)	78.5% (n = 408)
Cultured meat	0.2% (n = 1, in Singapore)	99.8% (n = 519)
Algae	91.9% (n = 478)	8.1% (n = 42)

**Table 3:** Intend to consume in the future.

Alternative Types	Never	Occasionally	Regularly
Plant-based meat	80.8% (n = 420)	17.7% (n = 92)	1.5% (n = 8)
Plant-based milk	55.0% (n = 286)	23.1% (n = 120)	21.9% (n = 114)
Insects	89.8% (n = 467)	10.2% (n = 53)	0% (n = 0)
Cultured meat	97.3% (n = 506)	2.7% (n = 14)	0% (n = 0)
Algae	49.4% (n = 257)	41.2% (n = 214)	9.4% (n = 49)

**Table 4:** No intention to consume alternative proteins again.

“I tasted a plant-based burger once, and it was not even closer to meeting my standards, and I was expecting a lot from it because my boss was telling me I should try them, and he is quite a foody. Now I know why he didn’t like it, as these “burgers” are not even closer to anything … to plants or meat. They are a greasy substance with ambiguous taste more unpleasant than pleasant and too salty.” (Female, born 1972)
“Don’t advertise me these veggie burgers. They are so unappetising not only with their pretentious look but especially with their unpleasant taste.” (Male, born 1989)
“I tried them, but honestly, I did not enjoy them. I will definitely not consume them in the future and will never give it to my kids.” (Female, born 1997)
“When I had a plant-based burger at a friend’s, it fell short of my expectations. It was overly fatty and greasy, and the worst thing was that it left a repulsive aftertaste that I remembered vividly for the next few days.” (Male, born 2000)

**Table 5:** Recognition of the benefits of plant-based milk.

“I was thinking that plant-based milks were created as there are people with lactose intolerance, but I am always puzzled why meats needed to be created at all.” (Male, born 1958)
“The only good option is perhaps plant-based milks and perhaps the insects, although we as consumers are not familiar with them.” (Female, born 1965)
“Plant-based milks are not so bad alternative milk options; they are great replacement of milk, especially for people with different allergies or intolerance.” (Female, born 1971)
“I am drinking regularly plant-based milks as I am lactose intolerant, but apart from the milks, I am not interested in any other options, not lab meat or insects.” (Male, born 1995)

**Table 6:** Plant-based milk consumption across generations.

Generation (Range of Birth Years Used)	Tried	Never Tried	Future Consumption			Total
			Occasional	Regular	Never	
Gen Z (1995–2010)	33.3% (n = 173)	0.8% (n = 4)	10.4% (n = 54)	11.0% (n = 57)	12.7% (n = 66)	34.0% (n = 177)
Millennials (1981–1994)	21.7% (n = 113)	1.2% (n = 6)	7.1% (n = 37)	7.5% (n = 39)	8.3% (n = 43)	22.9% (n = 119)
Gen X (1965–1980)	12.3% (n = 64)	11.5% (n = 60)	2.5% (n = 13)	1.5% (n = 8)	19.8% (n = 103)	23.9% (n = 124)
Baby Boomers (1946–1964)	7.5% (n = 39)	11.7% (n = 61)	3.0% (n = 16)	1.9% (n = 10)	14.2% (n = 74)	19.2% (n = 100)
Total:	74.8% (n = 389)	25.2% (n = 131)	23.1% (n = 120)	21.9% (n = 114)	55.0% (n = 286)	100% (n = 520)

**Table 7:** Plant-based milk consumption according to educational level.

Education	Tried	Never Tried	Future Consumption			Total
			Occasional	Regular	Never	
Year 10	82.6% (n = 19)	17.3% (n = 4)	47.8 (n = 11)	43.5% (n = 10)	8.7% (n = 2)	4.4% (n = 23)
High School	94.8% (n = 91)	5.2% (n = 5)	32.3% (n = 31)	38.5% (n = 37)	29.2% (n = 28)	18.4% (n = 96)
TAFE/College	75.7% (n = 84)	24.3% (n = 27)	53.2% (n = 59)	19.8% (n = 22)	27.0% (n = 30)	21.3% (n = 111)
Bachelor	73.2% (n = 145)	26.8% (n = 53)	7.6% (n = 15)	17.7% (n = 35)	74.7% (n = 148)	38.1% (n = 198)
Master	63.3% (n = 50)	36.7% (n = 29)	5.1% (n = 4)	12.7% (n = 10)	82.3% (n = 65)	15.2% (n = 79)
PhD	7.7% (n = 1)	92.3% (n = 12)	0% (n = 0)	0% (n = 0)	100% (n = 13)	2.5% (n = 13)
Total	74.8% (n = 389)	25.2% (n = 131)	23.1% (n = 120)	21.9% (n = 114)	55% (n = 286)	100% (n = 520)

**Table 8:** Concerns about contamination.

“There could be many issues [with] microbiological contamination because of the way they keep them in the supermarkets’ shelves.” (Male, born 1965)
“Any type of food contamination—chemical, microbial, physical, or allergenic—can occur with any food, including plant-based and other alternatives. I am particularly concerned about this possibility.” (Female, born 1972)
“I am concerned about alternative proteins’ storage processes. They can easily get contaminated being placed right next to meat in the supermarket shelfs … Contaminated food can result in food poisoning and, if not, diarrhea or vomiting.” (Female, born 1980)
“Yes, I am worried about food safety issues in the presence of microbiological contamination and potential presence of allergens with all the alternative proteins, not only plant-based, but algae and insects.” (Male, born 1999)

**Table 9:** Alternative proteins perceived positively.

“Lab-grown meat and milk could be better, as they, from what I read, are using no additives and there are not safety problems related to contamination as they are produced in the lab.” (Female, born 1970)
“No issues I can see; I actually believe they are good safe to consume choices and can provide valuable nutritional benefits.” (Female, born 1979)
“Not really, as I trust they are regulated and safe to consume, except for cultured meat and some types of algae as they are pretty new, and we are not yet familiar with them as products.” (Female, born 1981)
“Not sure if they have any issues I should be concerned about. I think they are good.” (Male, born 1983)

**Table 10:** Concern about artificial ingredients in alternative proteins.

“I can’t accept food made with any artificial ingredients. Artificial ingredients are increasing the risks of mental health disorders, cardiovascular diseases.” (Male, born 1968)
“There are so many additives to the alternative proteins. All these additives are required to improve the texture and taste of the fake meat, but in fact, they are creating health problems.” (Female, born 1977)
“I prefer real plant food, not processed food. All the alternative proteins are very heavily processed and contain many artificial ingredients.” (Male, born 1980)
“The world is changing, the foods we consume are changing, and now with the alternative proteins becoming a reality, we all should be scared that soon we will all be required to consume processed foods made from artificial ingredients. I am terrified. It’s getting worse than dealing with climate change.” (Female, born 2001)

**Table 11:** Concerns about the use of genetically modified organisms.

“Yes—not sure if it’s as good as meat protein. There is GMO products, especially soy.” (Male, born 1962)
“I am uncertain about any of the alternative proteins. They may be good, but I am not sure if they are as good as meat protein. Especially I am terrified that there is a GMO product, especially soy, that is used in making plant-based alternatives.” (Female, born 1974)
“No traceability of the ingredients. Made from soy, but what else? And what type of soy, GMO or something else? No one is telling you.” (Male, born 1977)
“Not that I am aware of what exactly alternative proteins area and what they are made from, perhaps some GMO-related issues with their ingredients should be taken into account when someone would like to consume it.” (Female, born 1999)

**Table 12:** Respondents’ concern about the long list of ingredients.

“The taste is okay at first, but then you have this feeling of being misled with the food content. They are mixed with preservatives, oils, natural or artificial colouring and seasonings, and other endless list of unknown ingredients.” (Female, born 1965)
“I don’t trust plant-based alternatives like plant-based burgers or sausages that usually attempt to replicate the look and taste of meat, and I am sure contain unhealthy ingredients. This is evident at the back of their packages.” (Male, born 1989)
“Absolutely worried due to the long ingredients list of these products. When you consume a real meat, you have beef, lamb, chicken, not 100 things listed as part of it.” (Male, born 1992)
“I have many concerns around the too many items listed in the ingredients list of every single plant-based burger, mince, sausages. Adding too many ingredients for sure is not making the end food product more palatable, but rather suspicious in term of its healthiness.” (Female, born 2002)

**Table 13:** Respondents’ concern regarding allergens.

“I worry about allergenic contamination in alternative protein production. It is quite a serious problem to be ignored by producers of these alternative proteins.” (Female, born 1971)
“I really enjoyed the insects … crickets and silk pupae I ate in China. Here, I used to buy cricket bars, which were with a nice nutty taste. I never thought that they may cause some food safety problems, but a friend of mine, another fan of insects’ bars, has developed an allergy to crustaceans. I am not buying these bars anymore, and I am warning all my friends about this.” (Female, born 1977)
“Allergy-related issues. As the ingredients are not clear, there may be some potential presence of allergens. I am allergic to a few things, and I don’t want to eat something that will cause me more allergic reactions.” (Male, born 1978)
“I think there are unforeseen safety issues—potential cross-contamination of allergens in the manufacturing processes.” (Female, born 2000)

**Table 14:** Respondents’ concern about health risks.

“When plant-based foods are cooked at high heat, they can be carcinogenic.” (Female, born 1971)
“There are so many additives to the alternative proteins. All these additives are required to improve the texture and taste of the fake meat, but in fact, they are creating health problems.” (Female, born 1978)
“Yes, I believe they can cause some cardiometabolic risk and can lead to problems with the human microbiome.” (Female, born 1980)
“Fake meat products, when cooked, can be carcinogenic.” (Male, born 1991)

**Table 15:** Respondents’ preference for traditional foods to avoid health risks.

“I am not keen, and also, I think that if you’re substituting these products for meat as a means of improving your health, like substituting with plant-based alternatives, you’ll probably get more value from eating good-quality lean meat than plant-based meat substitutes. They will be detrimental for your health due to their content.” (Male, born 1968)
“I think as consumer preferences start demanding certain ingredients over other ingredients, producers will turn their attention toward ingredient sourcing, particularly in niche commodity markets, to strike the right balance between quality and price. Right now, they are not nutritionally and price compatible to meat, and their quality is probably not what the consumers are expecting.” (Female, born 1977)
“Billions of development dollars have been spent trying to replicate the real thing, but me being a vegetarian [they] are out of their market efforts except for the plant-based milks. I am not consuming anything that resembles meat; plus, I am not sure if the solutions producers are giving us—lab meat, algae, insects—are healthy or nutritionally safe to consume.” (Female, born 1978)
“I don’t consume artificial stuff. I prefer normal and natural food.” (Male, born 2001)

**Table 16:** Concerns about dietary fibre and micro- and macronutrients.

“Alternatives are lacking essential nutrients. For instance, plant-based milks can be low in calcium, iodine, and vitamin B12, micronutrients, and macronutrients. The same applies for the other alternatives.” (Female, born 1979)
“I think artificial meats have some issues with micronutrients, dietary fibre. They are different compared to plant milks. No opinion about cultured meat. I never tried it, but I suspect it will have some substantial micronutrients issues. Algae will be okay in term of dietary fibres and, I think, micronutrients.” (Male, born 1986)
“I can say that usually plant-based foods are meant to provide good dietary fibre, which are not found in red meat, but they can’t provide the essential nutrients, micro- and macronutrients that red meat and other real meats provide. But the alternatives only imitate meat, and they are made of plants but not really plants. So, it’s quite concerning.” (Female, born 1998)
“Yes, I am concerned mainly because I am not sure if the alternative proteins and all these fake meats and algae and insects and lab meat and other alternatives are providing good nutrients and are a good protein source at all. I think they are at least half good as the real meat is.” (Female, born 2000)

**Table 17:** Respondents’ concern about high sodium content.

“Some plant-based meats contain relatively high amounts of salts, which may also be a health concern, as this can lead to high blood pressure, cardiovascular disease, kidney disease.” (Male, born 1980)
“Not healthy. Soy-based meat substitutes are very often high in salt and other unhealthy ingredients, including, I think, isolated soy proteins and other ingredients that may not be as healthy as they are presented. But the salt content is really of concern.” (Male, born 1986)
“I am a bit concerned as meat analogue can contain higher amounts of sodium as compared to animal meat.” (Female, born 1997)
“Contain unhealthy ingredients, high salt content. Also attempt to replicate the taste and texture of meat. I already stop consuming meat just occasionally when visiting friends, and in my opinion, I do not like to eat something that imitates meat and being five–six times saltier than the real meat. If I want to eat meat, I can always and will know what is included in the ingredients and will add as much salt as I like.” (Male, born 2002)

**Table 18:** Plant-based alternatives acceptable for occasional consumption.

“It feels like trying these alternative proteins plant-based, insect, algae, lab meat are part of following a modern trend rather than a genuine preference of us as consumers to consume. These alternatives don’t taste better than meat, but I do occasionally enjoy them like I enjoy consuming chicken nuggets or other processed food.” (Female, born 1967)
“I tried them as I was invited at a friend’s party. They were kind of fine, but I can perfectly live without touching them. I can eat them occasionally if needed though.” (Female, born 1972)
“While I’m sceptical about their nutrition and safety, they are acceptable to eat occasionally, just like other foods that aren’t completely safe.” (Male, born 1986)
“I have doubts about their nutrition quality and safety, but as with any food that’s not perfectly safe, they’re fine for occasional consumption.” (Male, born 2000)

**Table 19:** Transparency in labelling alternative proteins.

“These alternatives are well advertised, and I think not well explained in terms of nutrients. They are not equal to meat, and they aren’t healthy.” (Male, born 1980)
“I am a bit sceptical about alternative proteins and their quality nutrition-wise.” (Male, born 1981)
“These alternatives, I mean mainly the plant-based, provide nutrients that differ from those in meat products, but not all are safe to consume or even healthy.” (Female, born 1988)
“I don’t trust plant-based alternatives. I even think that vegetable protein-based products, like plant-based burgers or sausages that usually attempt to replicate the look, taste, and texture of meat, may contain unhealthy ingredients.” (Male, born 1999)

**Table 20:** Alternative proteins and masculinity.

“Lately there is a new wave of meatless meat, but many people like me like the real stuff, the juicy, bloody meaty taste and texture. It’s more manly and nutritiously wiser to stick to the real thing, not the fake meat. I feel the same in relation to other alternatives; insects and seaweeds are also not proper, nutritious, and safe food for males.” (Male, born 1970)
“My main concerns are not about the nutritional quality of these alternative food and drinks but more around my own manliness. I don’t know how to explain it, but currently, I feel these foods are against my existing and rather complex relationship with food and with my gender identity. I am finding alternative proteins not so manly for me to let them be part of my masculine identity.” (Male, born 1978)
“Nothing I can comment on. I am a man and will eat meat like a man, not the fake stuff.” (Male, born 1992)
“These alternatives are truly part of an agenda to operate us with food from our masculinity and by eating alternative proteins all the men to become not a real man.” (Male, born 2000)

**Table 21:** Generational differences.

Rejecting alternative proteins	“I worry because I can see the trend toward pushing all of us forward a societal transition toward more plant-based diets, but not toward consuming real plants and wholefood, but heavily processed alternatives and others, all with unclear safety and nutrition and health effects on humans.” (Male, born 1996)
	“I am not familiar with the plant-based meats. I never tried them and not willing to try. They look too fake.” (Female, born 2001)
	“These alternatives are having very complex formulations, which is a bit scary and perhaps not safe to consume.” (Female, born 2006)
	“Trying these alternatives is more about exploring new things rather than making them a regular habit. From a nutritional standpoint, I still think they fall short compared to meat.” (Male, born 1967)
Accepting alternative proteins	“Alternative proteins are still new. I believe if there are any issues, these will be sorted out by their producers.” (Male, born 1984)
	“It’s mostly a modern trend to try rather than truly enjoy. While the taste doesn’t compare to meat, it’s still food, and I find it enjoyable.” (Female, born 1987)

**Table 22:** Alternative proteins and sustainability.

“The only thing I consume is almond milk from time to time. I am not so serious about it, but sometimes, it gives me some sense of being sustainable in my food choices.” (Male, born 1969)
“I believe these food varieties are part of a big greenwashing campaign, part of a pretend sustainability agenda.” (Male, born 1999)
“I am afraid that these new foods that are emerging and present as good, sustainable, and ethical options for us. They are maybe sustainable and ethical, but not healthy.” (Male, born 2000)
“No, I understand that whilst it might be more sustainable and ethical, plant-based burger patties are not necessarily healthier than the meat equivalent. Claiming sustainability and ethical consumption is wrong when this is generally processed food that is not so safe to consume.” (Female, born 2000)
“I think it’s important to look at the bigger picture, not only the environmental sustainability and the greenhouse gas emissions, but also to the nutritional value of foods. I have no idea what the nutritional value of all alternative proteins are, except for insects and algae, as they are eaten for millions of years and still consumed around the world, so I assume it is good and sustainable.” (Female, born 2000)

## Data Availability

The original contributions presented in the study are included in the article/Appendix A, further inquiries can be directed to the corresponding author.

## Appendix A

**Table A1:** Survey questionnaire.

N	Questions	Type	Variations to Choose			
1	What does your diet look like?	Quantitative	Daily meat eater 4–5 times a week Flexitarian 2–3 times a week Occasional 1–2 times a week Vegetarian Vegan			
2	Have you ever consumed any alternative proteins, e.g., plant-based meat, plant-based milk, insect-based food, etc.?	Quantitative	Yes No (Go to) Maybe			
3	What do you think alternative proteins are?	Qualitative	Free answer			
4	What type of alternative proteins have you consumed?	Quantitative	Plant milk Plant-based meat Insects Algae			
5	Would you be willing to consume cultured meat (lab-grown meat) if available on the market?	Quantitative	Yes No Maybe			
6	You mentioned you have tried/have not tried (plant-based milk, plant-based meat, insects). Do you plan to consume (plant-based milk, plant-based meat, insects) in the future? Please choose what is applicable to you.	Quantitative	Plant milk	Plant-based meat	Insects	Algae
			Never Occasionally Regularly Unsure			
7	You mentioned you will be willing/will not be willing to try cultured meat, if available on the market. Do you plan to consume cultured meat in the future? Please choose what is applicable to you.	Quantitative	Plant milk	Plant-based meat	Insects	Algae
			Never Occasionally Regularly Unsure			
8	Why have you not tried any alternative proteins (plant-based milk, plant-based meat, insects), and what is the reason behind your decision?	Qualitative	Free answer			
9	When thinking of cultured meat, why are you not willing to try it? Please explain the reasons behind it.	Qualitative	Free answer			
10	When or after you have tried alternative proteins, have you had any concerns about the quality of these food/drinks?	Qualitative	Free answer			
11	Do you think there are some potential issues (e.g., health, nutrition, safety, etc.) that you believe should be considered in relation to alternative proteins? Can you explain?	Qualitative	Free answer			
12	What is your general opinion and attitude toward alternative proteins as part of our future food plate?	Qualitative	Free answer			

**Table A2:** Generation Z’s negative taste and texture opinion (excerpt).

1	“I tried a plant-based burger at a friend’s place, and it didn’t meet my expectations. It was too greasy and had an unpleasant aftertaste.” (Male, born 1997)
2	“These alternatives are with gross taste and too amalgamated texture. If their producers want to have a slight chance their product to be out there and to be met with interest from the consumers, they need to continue to improve their taste, texture, appearance, and especially nutritional quality.” (Male, born 1999)
3	“Fake meats’ texture can be unappealing, as it feels too soggy and wrong, making them less enjoyable to eat. At least for me.” (Female, born 2003)
4	“I tried some of the alternative proteins’ products like fake meats once out of curiosity. I wasn’t satisfied at all. I think the claims producers are making for these products are not substantiated, especially with the taste provided, their look, behaviour when cooking.” (Female, born 2001)
5	“I was put off by the taste of plant-based meat and the fact that it is artificially made; the texture wasn’t quite right and made me think that it is not necessarily a healthier alternative to regular meat despite what the producers claim.” (Female, born 2002)
6	“First, when I tried a plant-based burger, I felt really strange. The thing that others saw as meat imitation bothered me too much. The taste was disappointing. It was excessively fatty, oily, or more like greasy, and the worst part was the unpleasant aftertaste that lingered for days in my mouth. I still remember the awful taste.” (Male, born 2003)
7	“Fake meats have this mushy texture … soft and squishy, lacking any firmness or structure. No way this could replicate real meat.” (Male, born 2004))
8	“Texture is somehow unified, which could be not a bad thing if it was meat. I prefer not to need to taste it again.” (Female, 2000)
9	“I was at my cousin’s house, and there was my aunty who came up with the idea we to make spaghetti Bolognese with plant-based meat. You can’t imagine how this whole thing just collapsed into some strange substance which we fixed with sauce, but when we tasted, it was bloody problematic. It had so many issues. No one ate it, and we gave it to the dog, and Rickey didn’t want to even try it. I am not even kidding.” (Male, born 2002)
10	“Taste is what concerns me. It is not like the one of a conventional meat.” (Female, born 1999)
11	“Not impressed with plant-based meat. It feels somewhat like mashed potatoes or overcooked vegetables, where the food easily breaks apart and has a somewhat wet, pulpy consistency. I even think potatoes and vegetables are tastier than plant-based meat.” (Female, born 1998)
12	“Plant-based meats, in my opinion, often face criticism for their texture, which is mushy. Also, they have very unappetising appearance. I never was a fan of them.” (Male, born 1996)
13	“I don’t understand why they’re making these alternatives. I’ve never seen anyone buying them. The taste is strange, and the aftertaste is unpleasant.” (Male, born 2000)
14	“Awful choice of food. Fake meats are nothing closer to meat and not even near as good as meat taste wise; you could feel the chemicals or whatever they add in it.” (Female, born 2005)

## References

[1] Marinova D., Bogueva D. (2022). Food in a Planetary Emergency.

[2] Bryant C.J. (2022). Plant-based animal product alternatives are healthier and more environmentally sustainable than animal products. Future Foods.

[3] Ignaszewski E., O’Donnell M. (2024). Global Alternative Protein Trends to Watch. Good Food Institute. https://gfi.org/blog/global-alternative-protein-trends-to-watch/.

[4] Markets and Markets (2024). Protein Alternatives Market. https://www.marketsandmarkets.com/Market-Reports/alternative-protein-market-233726079.html?gad_source=1&gbraid=0AAAAADxY7SyPy1HGVh8pHOcQLcQeq8GXJ&gclid=Cj0KCQiAqL28BhCrARIsACYJvkckwyDo2VbiDKQ53YjbS3UZbzka7h_ZWRCk6gXpNhTektGn39w-FowaArENEALw_wcB.

[5] Food Frontier (2023). State of the Industry: Australia’s Plant-Based Meat Industry. https://www.foodfrontier.org/resource/2023-state-of-the-industry/.

[6] Food Frontier (2024). Frontier Consumer Survey 2024: Understanding Dietary Preferences and Changes in Australia. https://www.foodfrontier.org/resource/food-frontier-consumer-survey/.

[7] Whitton C., Bogueva D., Marinova D., Phillips C.J.C. (2021). Are we approaching peak meat consumption? Analysis of meat consumption from 2000-2019 in 35 countries and its relationship to Gross Domestic Product. Animals.

[8] Statisa (2024). Plant-Based Food in Australia—Statistics and Facts. https://www.statista.com/topics/8030/plant-based-food-in-australia/#topicOverview.

[9] Good Food Institute 2023 State of Global Policy: Public Investment in Alternative Proteins to Feed a Growing World. https://gfi.org/wp-content/uploads/2024/06/The-State-of-Global-Policy-on-Alternative-Proteins-2023.pdf.

[10] Good Food Institute (2024). Challenges and Breakthroughs: Contextualizing Alternative Protein Progress. https://gfi.org/blog/contextualizing-alternative-protein-progress/.

[11] Shah P., Geyik Ö., Archibald C.L., Hadjikakou M. (2024). Sustainable food choices require product-specific environmental footprints: The case of packaged food in Australia. Sustain. Prod. Consum..

[12] Healy J.D., Dhaliwal S.S., Pollard C.M., Sharma P., Whitton C., Blekkenhorst L.C., Boushey C.J., Scott J.A., Kerr D.A. (2023). Australian consumers’ attitudes towards sustainable diet practices regarding food waste, food processing, and the health aspects of diet: A cross sectional survey. Int. J. Environ. Res. Public Health.

[13] Good Food Institute Is Plant-Based Meat Ultra-Processed?. https://gfieurope.org/is-plant-based-meat-ultra-processed/.

[14] Chapman J. (2024). Processing the Discourse over Plant-Based Meat: Understanding Nuance in the “Ultra-Processed Food” Debate to Build Acceptance and Trust of Plant-Based Meat. https://media.churchillfellowship.org/documents/JChapman_-_Processing_the_discourse.pdf.

[15] Ford H., Zhang Y., Gould J., Danner L., Bastian S.E.P., Yang Q. (2024). Comparing motivations and barriers to reduce meat and adopt protein alternatives amongst meat-eaters in Australia, China and the UK. Food Qual. Prefer..

[16] Ford H., Zhang Y., Gould J., Danner L., Bastian S.E.P., Ford R., Yang Q. (2023). Applying regression tree analysis to explore willingness to reduce meat and adopt protein alternatives among Australia, China and the UK. Food Qual. Prefer..

[17] Shorten A., Smith J. (2017). Mixed methods research: Expanding the evidence base. Evid.-Based Nurs..

[18] Creswell J.W., Plano Clark V.L. (2011). Designing and Conducting Mixed Methods Research.

[19] Plano Clark V.L. (2016). Mixed methods research. J. Posit. Psychol..

[20] Lumivero Solutions to Empower Data Insights and Outcomes. https://lumivero.com/solutions/.

[21] LimeSurvey Welcome to LimeSurvey. The LifeSurvey..

[22] Lindemann N. (2024). What’s the Average Survey Response rate?: Tips for Consultants, Marketers, HR and Anyone Looking to Boost Data Collection. https://pointerpro.com/blog/average-survey-response-rate/.

[23] Australian Bureau of Statistics (ABS) Education and Training: Census. 28.06.2022. https://www.abs.gov.au/statistics/people/education/education-and-training-census/2021.

[24] Ong S. Singapore Doubles Down on Lab-Grown Meat as Silicon Valley Backs Off. 19 June 2024. https://restofworld.org/2024/lab-grown-meat-singapore/.

[25] Dietitians Australia (2022). Diet and Nutrition Health Advice: Plant-Based Milks. https://dietitiansaustralia.org.au/health-advice/plant-based-milks.

[26] Chronakis I.S., Madsen M.M., Phillips G.O., Williams P.A. (2011). Algal proteins. Handbook of Food Proteins.

[27] Zhou Y., Liu L., Li M., Hu C. (2022). Algal biomass valorisation to high-value chemicals and bioproducts: Recent advances, opportunities and challenges. Bioresour. Technol..

[28] Bogueva D., Marinova D., Bryant C. (2022). Meat me halfway: Sydney meat-loving men’s restaurant experience with alternative plant-based proteins. Sustainability.

[29] Velzeboer R., Li E., Gao N., Sharp P., Oliffe J.L. (2024). Masculinity, meat, and veg*nism: A scoping review. Am. J. Mens. Health.

[30] Bryant C., Dillard C. (2019). The impact of framing on acceptance of cultured meat. Front. Nutr..

[31] Hefferon K.L., De Steur H., Perez-Cueto F.J.A., Herring R. (2023). Alternative protein innovations and challenges for industry and consumer: An initial overview. Front. Sustain. Food Syst..

[32] Jafarzadeh S., Qazanfarzadeh Z., Majzoobi M., Sheiband S., Oladzadabbasabad N., Esmaeili Y., Barrow C.J., Timms W. (2024). Alternative proteins; A path to sustainable diets and environment. Curr. Res. Food Sci..

[33] Malila Y., Owolabi I.O., Chotanaphuti T., Sakdibhornssup N., Elliott C.T., Visessanguan W., Karoonuthaisiri N., Petchkongkaew A. (2024). Current challenges of alternative proteins as future foods. NPJ Sci Food.

[34] Onwezen M.C., Bouwman E.P., Reinders M.J., Dagevos H. (2021). A systematic review on consumer acceptance of alternative proteins: Pulses, algae, insects, plant-based meat alternatives, and cultured meat. Appetite.

[35] MedlinePlus (2023). Lactose Intolerance. https://medlineplus.gov/genetics/condition/lactose-intolerance/#frequency.

[36] Dairy Australia Is it True That Most Asian People Are Lactose Intolerant?. https://www.dairy.com.au/dairy-matters/you-ask-we-answer/yawa-29---is-it-true-that-most-asian-people-are-lactose-intolerant.

[37] Dynan N. Reducing the Personal and Health Impacts on Those Suffering Lactose Intolerance. The Good Nutrition Co. 2020. https://member.dietitiansaustralia.org.au/Common/Uploaded%20files/DAA/Resource_Library/2020/Lactose_Intolerance_Ndynan.pdf.

[38] Ritchie H. (2022). Dairy vs. Plant-Based Milk: What Are the Environmental Impacts? Our World in Data. https://ourworldindata.org/environmental-impact-milks.

[39] Siddiqui S.A., Alvi T., Sameen A., Khan S., Blinov A.V., Nagdalian A.A., Mehdizadeh M., Adli D.N., Onwezen M. (2022). Consumer acceptance of alternative proteins: A systematic review of current alternative protein sources and interventions adapted to increase their acceptability. Sustainability.

[40] Birch D., Skallerud K., Paul N. (2019). Who eats seaweed?: An Australian perspective. Int. Food Agribus. Mark..

[41] Baker M.A., Shin J.T., Kim Y.W. (2016). An exploration and investigation of edible insect consumption: The impacts of image and description on risk perceptions and purchase intent. Psychol. Mark..

[42] Verneau F., La Barbera F., Kolle S., Amato M., Del Giudice T., Grunert K. (2016). The effect of communication and implicit associations on consuming insects: An experiment in Denmark and Italy. Appetite.

[43] Sogari G., Bogueva D., Marinova D. (2019). Australian consumers’ response to insects as food. Agriculture.

[44] Sogari G., Riccioli F., Moruzzo R., Menozzi D., Tzompa Sosa D.A., Li J., Liu A., Mancini S. (2023). Engaging in entomophagy: The role of food neophobia and disgust between insect and non-insect eaters. Food Qual. Prefer..

[45] Bogueva D., McClements D.J. (2023). Safety and nutritional risks associated with plant-based meat alternatives. Sustainability.

[46] Mihalache O.A., Dellafiora L., Dall’Asta C. (2023). Assessing the Mycotoxin-related Health Impact of Shifting from Meat-based Diets to Soy-based Meat Analogues in a Model Scenario Based on Italian Consumption Data. Expo Health.

[47] Messina M., Duncan A.M., Glenn A.J., Mariotti F. (2023). Perspective: Plant-based meat alternatives can help facilitate and maintain a lower animal to plant protein intake ratio. Adv. Nutr..

[48] Amyoony J., Moss R., Dabas T., Gorman M., Ritchie C., LeBlanc J., McSweeney M.B. (2023). An investigation into consumer perception of the aftertaste of plant-based dairy alternatives using a word association task. Appl. Food Res..

[49] Grossmann L., Kinchla A.J., Nolden A., McClements D.J. (2021). Standardized methods for testing the quality attributes of plant-based foods: Milk and cream alternatives. Compr. Rev. Food Sci. Food Saf..

[50] Appiani M., Cattaneo C., Laureati M. (2023). Sensory properties and consumer acceptance of plant-based meat, dairy, fish and eggs analogs: A systematic review. Front. Sustain. Food Syst..

[51] Smith V., Smith R. (2024). 20 Vegan Athletes You Didn’t Know Ate a Plant-Based Diet. https://www.veganfoodandliving.com/features/vegan-athletes-plant-based-diet/.

[52] Kraak V.I. (2022). Perspective: Unpacking the wicked challenges for alternative proteins in the United States: Can highly processed plant-based and cell-cultured food and beverage products support healthy and sustainable diets and food systems?. Adv. Nutr..

